# Pattern Classification Using Quantized Neural Networks for FPGA-Based Low-Power IoT Devices

**DOI:** 10.3390/s22228694

**Published:** 2022-11-10

**Authors:** Manas Ranjan Biswal, Tahesin Samira Delwar, Abrar Siddique, Prangyadarsini Behera, Yeji Choi, Jee-Youl Ryu

**Affiliations:** 1 Department of Intelligent Robot Engineering, Pukyong National University, Busan 48513, Republic of Korea; 2 Department of Electrical and Computer Engineering, University of Waterloo, Waterloo, ON N2L 3G1, Canada

**Keywords:** internet of things (IoT), convolutional neural network (CNN), quantized neural network (QNN), binary neural network (BNN), FPGA, pattern recognition

## Abstract

With the recent growth of the Internet of Things (IoT) and the demand for faster computation, quantized neural networks (QNNs) or QNN-enabled IoT can offer better performance than conventional convolution neural networks (CNNs). With the aim of reducing memory access costs and increasing the computation efficiency, QNN-enabled devices are expected to transform numerous industrial applications with lower processing latency and power consumption. Another form of QNN is the binarized neural network (BNN), which has 2 bits of quantized levels. In this paper, CNN-, QNN-, and BNN-based pattern recognition techniques are implemented and analyzed on an FPGA. The FPGA hardware acts as an IoT device due to connectivity with the cloud, and QNN and BNN are considered to offer better performance in terms of low power and low resource use on hardware platforms. The CNN and QNN implementation and their comparative analysis are analyzed based on their accuracy, weight bit error, RoC curve, and execution speed. The paper also discusses various approaches that can be deployed for optimizing various CNN and QNN models with additionally available tools. The work is performed on the Xilinx Zynq 7020 series Pynq Z2 board, which serves as our FPGA-based low-power IoT device. The MNIST and CIFAR-10 databases are considered for simulation and experimentation. The work shows that the accuracy is 95.5% and 79.22% for the MNIST and CIFAR-10 databases, respectively, for full precision (32-bit), and the execution time is 5.8 ms and 18 ms for the MNIST and CIFAR-10 databases, respectively, for full precision (32-bit).

## 1. Introduction

Deep learning has enabled artificial intelligence (AI) to quickly progress in recent years. The convergence of AI and the internet of things (IoT) is redefining the way industries, businesses, and technologies function. IoT can be used in various applications of automation with less or no human intervention. AI makes it possible for machines to learn from experience, adjust to new inputs, and perform human-like tasks. One of the primary objectives of AI is pattern classification and recognition. One of the ways that pattern recognition is carried out is through using rectangular windows followed by a classification process [1,2]. Numerous machine learning algorithms have been developed that play a vital role in pattern recognition. There are numerous algorithms, such as support vector machines (SVM), stochastic gradient descent (SGD), etc., for feature extraction. However, a typical convolutional neural network (CNN) has the ability to learn, adapt, and extract features automatically from existing databases [3,4]. In short, CNNs are the the popular method for any pattern classification or object detection algorithm [5,6]. However, they are computationally intensive and are difficult to deploy in any mobile interface device. To develop the performance of feature representation, the design induces more complexity, and their deployment on mobile platforms can be challenging. So, the need for a lightweight network configuration, without decreasing the accuracy of the system, remains [7].

**Figure 1:**
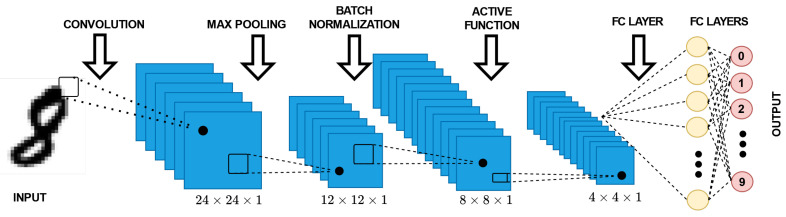
Architecture of a CNN.

A typical architecture of a CNN can be seen in Figure 1. In general, a CNN contains three basic layers. The first layer is the convolutional layer [8], the second is the pooling layer followed by the batch normalization layer, and the third is an output later, which is a fully connected layer. At each layer, the features are classified, and subsequently the information is passed on to the next layer. The binary neural network (BNN) approach is similar to CNN but provides better storage and memory capacity.

**Figure 2:**
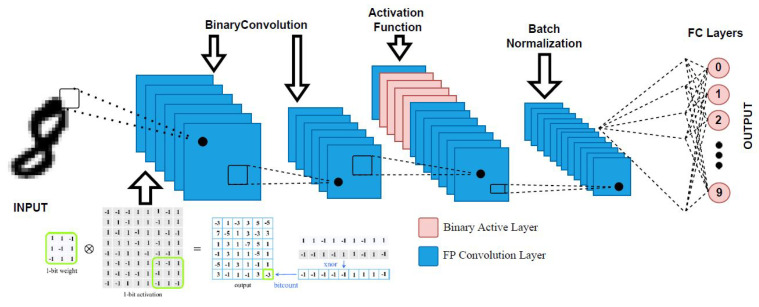
Architecture of a BNN.

Another approach is the BNN. This approach is a type of DNN processor that provides better storage space in terms of computing and memory usage while having a marginal tradeoff with accuracy. For a detailed performance comparison, some works have analyzed the BNN approach using the processor model of an infrared spectrum (IR) human-based image recognition application. In one work, algorithm analysis was performed for a 32-bit DNN using various model parameters, such as the kernel number and synaptic connection [9]. In this, the system structure was applicable for a BNN, such as an application-specific integrated circuit or a field programmable gate array (FPGA). The local storage was leveraged; that is, the full storage in the weights of BNN was used to speed up the process. Recently, object detection was performed using a hybrid convolutional recurrent network [1]. This work used the BNN to compare its performance against a GPU. A BNN’s architecture can be seen in Figure 2. Here, the input image is passed to the binary convolution layer. The features are scaled down before being passed to the next activation function for the classification of the pattern in another format, followed by the batch normalization layer. The output is a discovered pattern or object.

**Figure 3:**
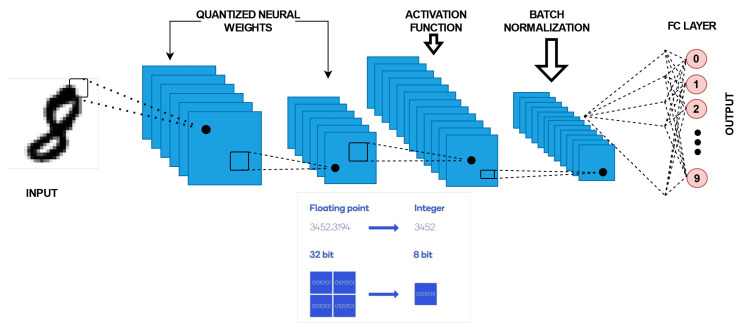
Architecture of a QNN.

**Figure 4:**
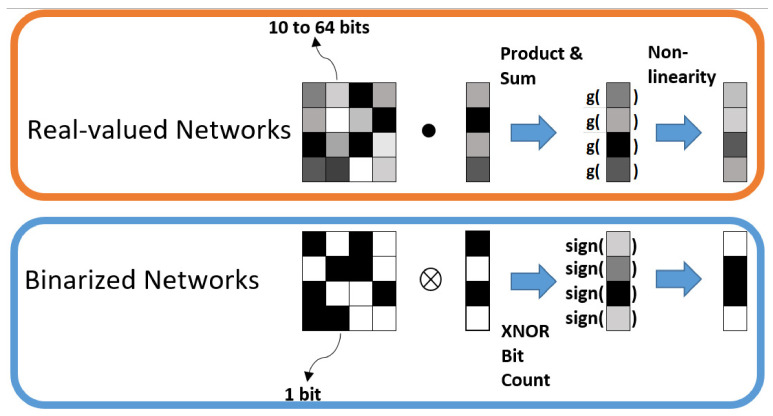
The difference weight implementation and distribution between a full precision neural network and a binarized neural network [14].

Another form of NN is the quantized neural network (QNN). QNNs are low-precision neural networks and have similar performance to a CNN while safeguarding the network structure. A BNN is also a form of amQNN. QNN consists of a quantization layer, multiply accumulation (MAC) layer, and a batch normalization layer. Although the storage and computation costs are greatly reduced, there is data loss due to quantization errors, which occurs under tradeoff conditions. A suitable quantization architecture should be able to store useful information in continuous variables and is critical to network performance. Figure 3 shows a QNN architecture. The network layer contributes differently to the overall performance, each with different sensitivities to size. As the network propagates forward, the variability of the hierarchical functions subsequently rises. In the shallow layer, the internal features are dispersed over multiple complexes. To obtain the functions as in [10,11], we need to have accurate neurons. At deeper layers, the coarse convolution filter can distinguish the previous local features [12]. Thus, parameter accuracy can be flexibly designed using network configuration and hierarchical feature distribution [13]. The difference in weight implementation and distribution between a full precision neural network and a BNN [14] is shown in Figure 4. While the full precision values can vary from 10 to 64 bits or even more, the BNN has a 1 bit memory stack.

There were various methods of training and quantization described in [15,16]. In most methods, the major challenges were the following: (a). In [15], the authors used the Pascal VOC database, with 2000 iterations for training purpose. This consumed a lot of storage space and I/O resources. (b). Extracting each range’s suggested feature individually wasted a lot of resources because the DNN function’s sharing feature was not used.

The major contributions of this paper are as follows:

- The paper analyzes the effect of pattern classification on various DNN classifiers on a IoT based hardware platform, including CNN, QNN, and BNN, and their implementation on an FPGA based IoT platform.
- The hardware used in the work is low power, compatible with Python, and mobile, which can also connect to the cloud, making it more compatible with IoT devices.
- Using the architecture of a BNN, we can reduce the computing time without much variation in the accuracy.
- We use the MNIST and CIFAR-10 databases for our analysis in terms of the prediction accuracy, weight bit error rate, RoC curve, and execution time on the PYNQ Z2 FPGA board.

In the remainder of this paper, the related work and a literature survey are presented in Section 2; the background and the system concept for an IoT-based pattern detection implemented in the PYNQ Z2 FPGA ZYNQ board are described in Section 3, based on CNN, QNN, and BNN DNN; Section 4 presents the results and discussion, and Section 5 concludes this work.

## 2. Related Work

The recent works on pattern recognition are described as per the CNN, QNN, and BNN works in the FPGA- and IoT-based platforms.

One of the earlier works on BNN was proposed by Austin et al. [17]. The author described the implementation of a binary neural network on a hardware platform. The research showed that training and implementation was 200 times faster than that of a current workstation. However, with the advent of modern computing platforms, numerous techniques have improved this over time.

A recent work by Li et al. [9] proposed a fixed sign binary neural network for the IoT. They demonstrated that their FSB algorithm achieved almost full precision results using CIFAR-10 and an imageNet database. They showed that the resource constrained IoT platforms could use BNN along with their proposed algorithm for better accuracy and faster computation.

A VLSI-implemented BNN for an embedded vision-based IoT was proposed by Kumar et al. [18]. They proposed binary weighted convolutional neural networks and realized them on an FPGA, which helped in the case of low energy resources.

An IoT application for remote sensing application was proposed by Reiter et al. [19]. The main motive to develop the application was to reduce the bandwidth of expensive earth observation images during capture and transmission. The authors developed a BNN and QNN based real-time remotely sensed cloud detection using FPGA.

Another work on QNN was proposed by Chang et al [20]. To counter the complex memory and computational complexity involved in full precision CNN, a new method called CLIP-Q was proposed. The authors developed a software and hardware codesigned platform and tested it on the CIFAR-10 and CIFAR-100 datasets. A similar kind of work was proposed by [21] for quantizing the weights of pretrained neural networks, which was used for multilayer perceptron (MLP) and CNN. The authors used a method to quantize each neuron or hidden unit using a greedy path-following algorithm. They used the MNIST, CIFAR-10, and VGG16 datasets. The methods were more computationally efficient, and the quantization error also decayed with the width of the NN layer.

Due to the difficulty of training a mixed precision model and the vast space of all possible bit quantizations, finding an optimal mixed precision model that can preserve accuracy while satisfying specific constraints on the model size and computation is extremely difficult. For this, Yu et al. [22] proposed a novel soft Barrier Penalty-based NAS (BP-NAS) for mixed precision quantization. This ensured that all the searched models were within the valid domain defined by the complexity constraint and thus could return an optimal model under the given constraint by conducting a single search.

Similarly, Chu et al. [15] proposed a mixed-precision QNN with progressively decreasing bitwidth. For feature representation, a bitwidth assignment heuristic based on quantitative separability was provided. Several common CNNs, such as AlexNex, ResNet, and Faster R-CNN, were quantized using the proposed mixed-precision method. Bartan and Mert [23] presented a convex optimization strategy for training quantized NNs with polynomial activations; hidden convexity in two-layer neural networks, semidefinite lifting, and Grothendieck’s identity were all used for their method.

For network compression, QNN can be an efficient approach that can be implemented on FPGAs. Chen et al. implemented n-bit QNNs [24]. The authors’ work was able to show similar accuracy when they used Resnet, Densenet, and Alexnet. The experimental work was conducted on an Xilinx ZCU102 platform.

Due to significant losses from the XNOR operations due to binary mapping, Wang et al. [25] proposed a channel-wise interaction-based binary convolutional neural network learning method (CI-BCNN) for efficient inference. The authors mined the channel-wise interactions by reinforcement learning. For this purpose, they used the CIFAR-10 and ImageNet datasets.

With all the scenarios for recently developed DNN classifiers, the challenge for a simple and readily available computer vision application platform for IoT application still remains. With the demand for low power and higher computing efficiency, the need to use an efficient algorithm for various IoT based applications is always a challenge.

## 3. Background and System Concept

Pattern classification and subsequent object recognition is the process of recognizing objects and classifying patterns in images. In the case of digital images and video, object recognition is a computing technology related to computer vision that includes image processing that handles the recognition of instances of specific semantic objects (people, buildings, cars, etc.).

Patterns or input images are recognized and categorized according to the CNN, QNN, and BNN methods. In Figure 1, the input image is collected from the MNIST and CIFAR-10 datasets. The image is first reprocessed for noise removal, which also includes various undesirable regions of the target image. The features of the datasets are classified using the DNN techniques of CNN, BNN, and QNN. In the real-world scenario, the DNN classifier plays a vital role in computer vision, natural language processing, and speech recognition. However, one of the challenges is that DNNs consume large amounts of memory and perform at a high computational cost. This is a great disadvantage to any mobile and compact devices and can greatly impact the battery life.

### 3.1. Convolution Neural Network (CNN)

**Figure 5:**
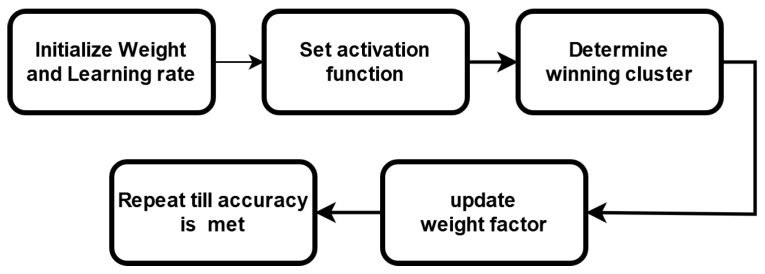
Block diagram of a CNN for pattern classification.

The CNN classifier for pattern classification can be described as shown in Figure 5. The CNN classifier contains three major blocks: (a) a convolution layer, (b) a pooling layer, and (c) an activation layer.

The convolution layer is the main unit of any CNN process. This is the first layer, which takes the input. This layer extracts the feature from the input image. It has three main operations, standard convolution, extended convolution, and transpose convolution.

### 3.2. Binarized Neural Network (BNN)

**Figure 6:**
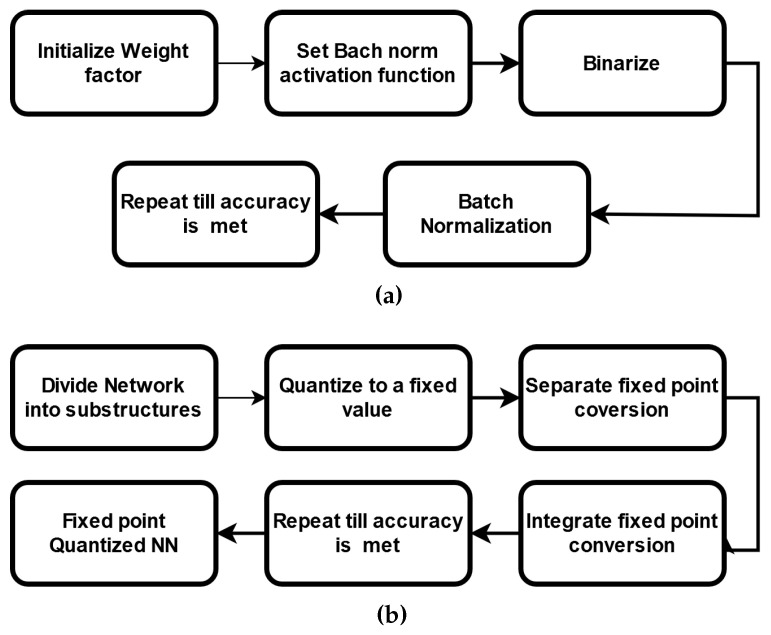
Block diagram of (**a**) BNN and (**b**) QNN for pattern recognition.

DNNs are computationally expensive and memory intensive for training and storing models. For miniature devices such as IoT devices, this poses a challenge. The binary neural network (BNN) is bounded by the set [+1, −1] [26]. BNNs require much less memory, which is why they are preferred for low-power devices [27]. A single-bit of binary data occupies only 1/32 times of the memory needed for a 32-bit display and has 32 times less memory access. A BNN’s architecture is almost identical to that of a CNN; the only difference is that all of its parameters are binarized to either +1 or −1. The −1 is encoded as 0, and +1 is encoded as 1. While using the CIFAR-10 dataset, the BNNs, constructed in the beginning, lost roughly 3% of their accuracy. Binary convolution is easy, as it is computed as 0, 1, and its hardware execution is as easy since it uses a simple XNOR function with a smaller portion. This leads to a lower hardware cost for binary arithmetic. Figure 6a roughly describes the flowchart block of a BNN.

### 3.3. Quantized Neural Network (QNN)

**Figure 7:**
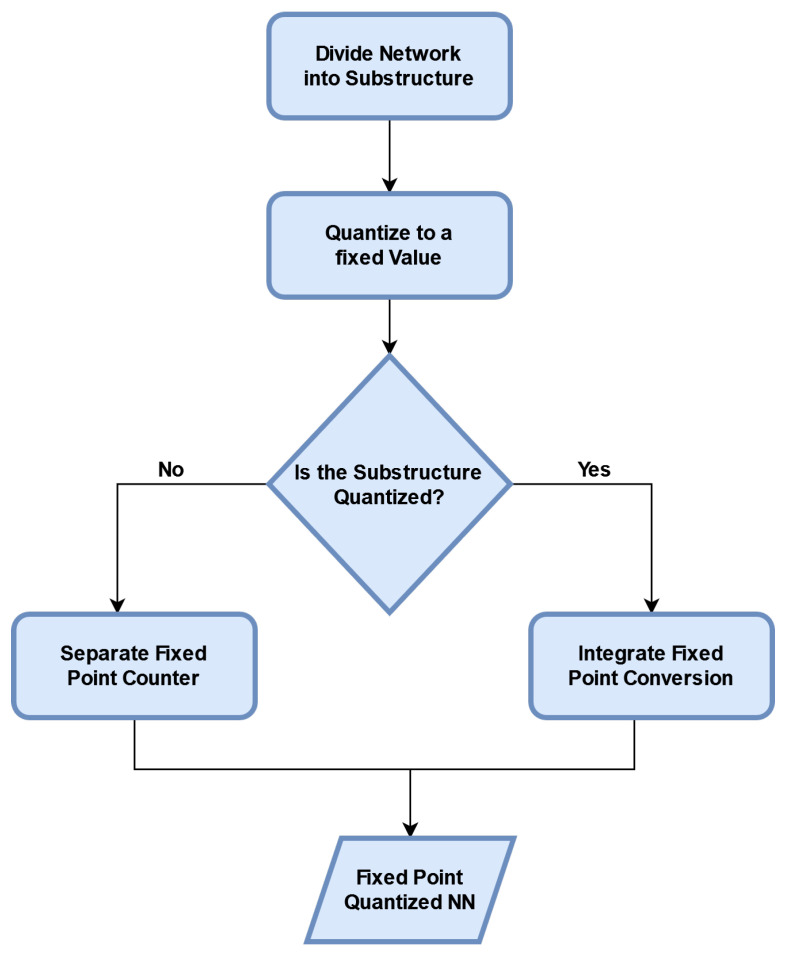
Flow diagram of a QNN for object detection.

As mentioned, QNNs are low-precision neural networks and can perform similarly to CNN while safeguarding the network structure. A BNN is also a form of a QNN. It defines the model parameters that are conserved by using a smaller bit width; moreover, the model size is reduced several times. QNN consists of a quantization layer, a multiply accumulation (MAC) layer, and a batch normalization layer. Although the storage and computation costs are greatly reduced, data loss due to quantization errors occurs under tradeoff conditions. A suitable quantization architecture should be able to store useful information in continuous variables and is critical to network performances. Figure 7 describes the typical blocks of a QNN flowchart.

For quantization, to convert from a floating point to fixed point, we need to find the required number of bits m to represent the unsigned integer part:

(1) $$m=1+\left[\log_{2}(\underset{1<i<N}{max}|x_{i}\left|\right)\right].$$

In the process of quantizing a network, the network can be deployed to the IoT device. Deployment includes:

- Exporting DNN weights and encoding them in a suitable format for on-target inference.
- Creating an inference program based on the DNN’s architecture.
- Compiling the program.
- Uploading a weighted inference program to the IoT platform.

**Figure 8:**
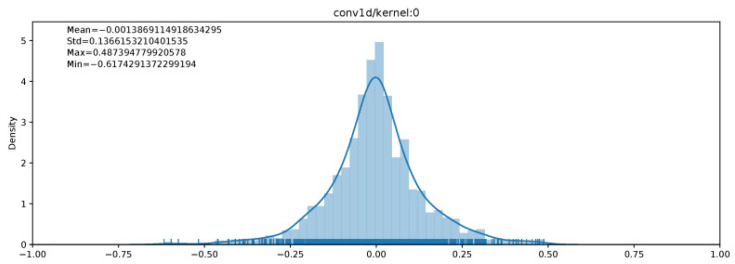
Weight distribution in a convolutional neural network kernel layer [28].

Figure 8 demonstrates the distribution for a convolutional kernel layer.

### 3.4. Overall Process for Deploying the Algorithms

1. Import the MNIST and CIFAR-10 database sets.
2. Set the parameters required for training.
3. Load a DNN classifier, i.e., CNN, BNN, or QNN.
4. Perform DNN on the CPU and also on the FPGA-based IoT device.
5. Observe and analyze the parametric results.

## 4. Results and Discussion

**Figure 9:**
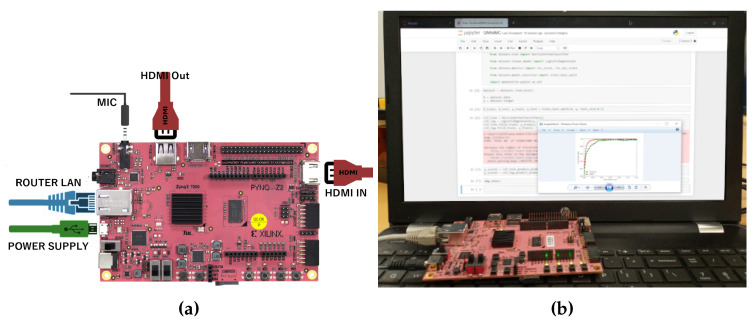
The PYNQ Z2 Xilinx board and its operational setup. (**a**) FPGA Xilinx 7200 Pynq Z2 board; (**b**) setup with the RoC curve for the CIFAR-10 and MNIST for CNN.

For the purpose of analysis and validation, we used an FPGA board, which acted as a IoT platform. The FPGA board is a Xilinx 7020 Zynq board, which can be connected through the cloud and has low power for its operation (5–7 V DC). The board is also compatible with Python, which is suitable for the purpose of analysis and experimentation. Figure 9 shows the hardware board used for our simulation and analysis. The MNIST and CIFAR-10 databases were used for the purpose of analysis and experimentation. The experimentation was performed on a CPU and then implemented on the FPGA hardware. The PC had an intel Core i5-4590 3.30 GHz CPU, 16.0 GB RAM, and a Windows 10 Pro 64-bit OS. Python was used for the evaluation and analysis. The accuracy, execution time, weight bit error rate, and RoC curve were analyzed for the DNN classifiers. Subsequently, the algorithms were implemented on an FPGA-based IoT boar, which is Python compatible, which acted as the IoT device. It was a ZYNQ 7020 board with 512 DDR3. The board is very mobile and qualifies as an IoT device with all the features; it can be connected to the cloud, has a lower power usage, and can be customized as an remote sensor for multipurpose functionality [29].

### 4.1. MNIST Dataset

MNIST is an abbreviation for the Modified National Institute of Standards and Technology Dataset. This is a collection of 60,000 tiny square grayscale photos of handwritten basic digits ranging from 0 to 9 [30]. Several standard databases have evolved to aid machine learning and pattern recognition research, in which handwritten numbers are preprocessed, including hashing and normalization, to allow researchers to compare the recognition results of various approaches on a common foundation. The MNIST database for handwritten digits is open source and has become a standard for quick testing of machine learning methods.

### 4.2. CIFAR-10

The CIFAR-10 is a dataset for image classification developed by the Canadian Institute for Advanced Research (CIFAR). There are 60,000 32 × 32 color pictures in the dataset, with 50,000 for training and 10,000 for testing. Planes, vehicles, birds, cats, deer, dogs, frogs, horses, boats, and trucks are divided into ten categories [31].

### 4.3. Performance Measures

The following performance parameters were analyzed:

- Prediction accuracy
- Weight bit error
- RoC curve
- Power consumption
- Execution time.

**Figure 10:**
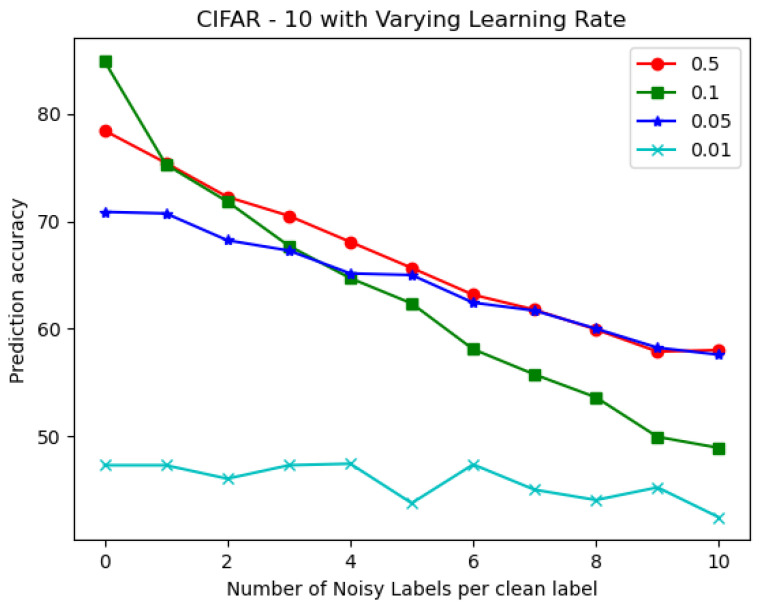
Variation in accuracy with respect to the LR in the CIFAR-10 for the QNN.

The FPGA-based IoT device is shown in Figure 9a, and the setup is shown in Figure 9b. Figure 10 shows the validation accuracy of the CIFAR-10 with the varying learning rates. The learning rate should be chosen optimally. The learning rate with 0.01 showed a low prediction rate, and 0.5 had a high prediction rate for QNN, but it decreased sharply with noisy labels.

Table 1, Table 2 and Table 3 show the execution times for various quantization levels from the CPU and the ZYNQ board with integer values of 8, 16, and 32 bits, respectively, with respect to the MNIST database, showing the performances for various quantization levels.

Similarly, Table 4 and Table 5 shows the inference times on the CPU and FPGA, respectively. The accuracy on the CPU for the MNIST 32 bit was 98.3%, and its accuracy was 95.5 % on the ZYNQ FPGA board.

The power consumption of the FPGA shown in Table 6 was the peak value reached during the run time.

**Figure 11:**
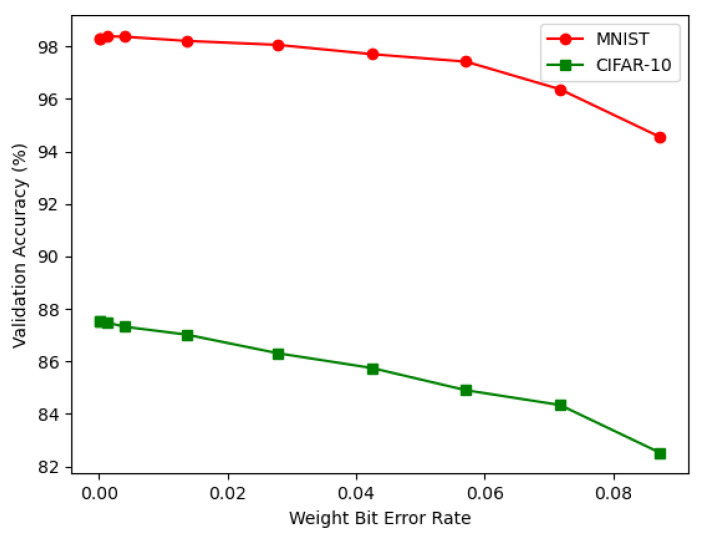
Weight bit error rate for the validation accuracy for the MNIST and CIFAR-10 databases.

The weight bit error rate for the validation accuracy is shown in Figure 11.

**Figure 12:**
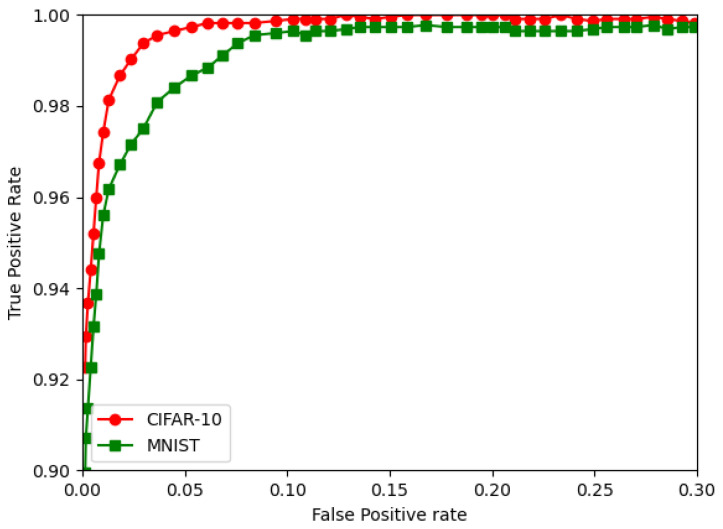
The RoC curves for the CIFAR-10 and MNIST for the CNN.

**Figure 13:**
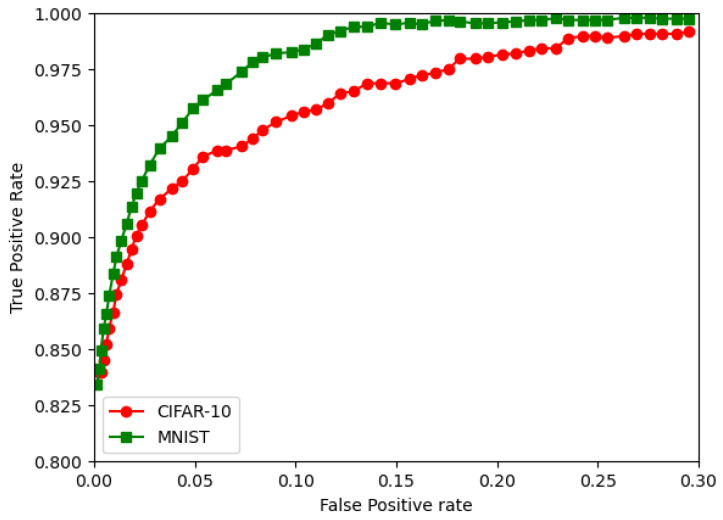
The ROC curves for the CIFAR-10 and the MNIST for the BNN.

**Figure 14:**
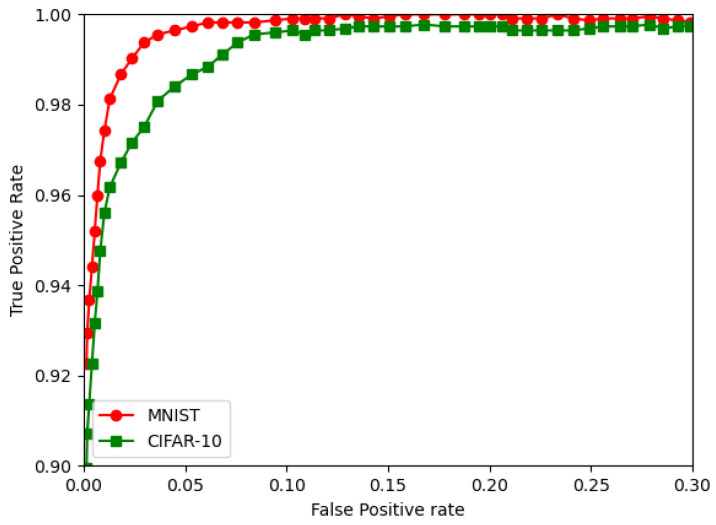
The ROC curves for the CIFAR-10 and the MNIST for the QNN.

The RoC curves for the MNIST and CIFAR are shown in Figure 12, Figure 13 and Figure 14. They show the FPGA for the CNN, followed by the BNN and QNN, respectively.

## 5. Conclusions

A detailed analysis of two pattern classification classes was implemented on low power IoT-based FPGA hardware. The challenge with the DNN-based pattern classification method can be overcome by using a quantized neural network; furthermore, BNN can also be used. The work was performed on an XILINX Zynq 7020 series Pynq board, which served as the hardware platform. The MNIST and CIFAR-10 databases were considered for simulation and experimentation. In this work, the CNN, QNN, and BNN classification algorithm models for the MNIST and CIFAR-10 databases were successfully implemented on an IoT-based hardware platform. The DNN classifier model was implemented in Python and analyzed for various evaluation parameters. These included the prediction accuracy, weight bit error rate, ROC curve, and execution time. The results showed that the BNN and QNN could be efficiently implemented and operated on mobile hardware devices such as the one used in this work, i.e., the ZYNQ Z2 board, which is an FPGA-based device that can be used as an IoT device. The analysis from the experiments performed using the FPGA-based hardware showed that the QNN, including the BNN, for pattern recognition application for IoT applications can be used in IoT based applications including in real time. This can be used for the future scope of the work. Most importantly, the QNN replaced the conventional CNN, reducing the device’s memory usage and computational resources.

## Figures and Tables

**Table 1:** Execution time for different platforms and models, integer 8.

Model	CPU	ZYNQ Z2 Board
MNIST	0.3 ms	2.9 ms
CIFAR-10	20 ms	160 ms

**Table 2:** Execution Time for different platforms and models, integer 16.

Model	CPU	ZYNQ Z2 Board
MNIST	0.6 ms	4.7 ms
CIFAR-10	24 ms	174 ms

**Table 3:** Execution Time for different platforms and models, integer 32.

Model	CPU	ZYNQ Z2 Board
MNIST	0.7 ms	5.8 ms
CIFAR-10	27 ms	180 ms

**Table 4:** Inference Times on the CPU.

Dataset	Inference Time (s)	Accuracy (%)
MNIST (FP 32)	79.56 × $10^{-3}$	98.3
CIFAR-10 (FP 32)	385.74 × $10^{-3}$	82.3

**Table 5:** Inference Times on the FPGA.

Dataset	Inference Time (s)	Accuracy (%)
MNIST (FP 32)	8.41 × $10^{-6}$	95.5
CIFAR-10 (FP 32)	327.74 × $10^{-6}$	79.22

**Table 6:** Peak value of the power consumption of the FPGA reached during the run time, FP32—Full precision 32 bit, INT 8—8 bit integer.

Platform	Precision	Power Consumption
MNIST FPGA	FP 32	2.70 W
MNIST FPGA	INT 8	2.54 W
CIFAR-10 FPGA	FP 32	2.94 W
CIFAR-10 FPGA	INT 8	2.66 W

## Data Availability

Not applicable.
